# Paraneoplastic Pemphigus in a Patient With Pheochromocytoma: A Report of a Rare Case

**DOI:** 10.7759/cureus.79459

**Published:** 2025-02-22

**Authors:** Zhong-Zhou Huang, Ming-Jie He, Ping Huang, Min-Qing Luo, Dan Hong, Sha Lu, Qing Guo, Liangchun Wang, Hui Xiong

**Affiliations:** 1 Department of Dermatology, Sun Yat-sen Memorial Hospital, Sun Yat-sen University, Guangzhou, CHN; 2 Department of Epidemiology and Public Health, School of Public Health, Sun Yat-sen University, Guangzhou, CHN

**Keywords:** autoantibody, autoantigen, autoimmune diseases, desmoglein, methotrexate, methylprednisolone, paraneoplatic pemphigus, pemphigus vulgaris, pheochromocytoma, rituximab

## Abstract

This report details a rare case of paraneoplastic pemphigus (PNP) associated with pheochromocytoma. The patient presented with prominent dermatological manifestations, including erythema, vesicles, and erosions. During hospitalization, diagnostic imaging revealed a retroperitoneal mass, which was subsequently surgically removed. A comprehensive diagnostic workup, including CT, MRI, histopathological analysis, and direct immunofluorescence examination, was conducted. Postoperative management combined with pharmacological intervention led to significant clinical improvement. This case highlights the critical importance of considering PNP in the differential diagnosis of pheochromocytoma, particularly in patients presenting with complex autoimmune manifestations. The findings underscore the necessity for early diagnosis and prompt therapeutic intervention in such cases. Additionally, this report emphasizes the need for further investigation into the clinical spectrum, genetic associations, and underlying mechanisms of this rare disease association to enhance diagnostic accuracy and therapeutic outcomes.

## Introduction

Paraneoplastic pemphigus (PNP) is a rare autoimmune blistering disorder that often involves the skin, mucous membranes, and multiple organs, with a high mortality rate of up to 90% [1]. The disease was first identified and characterized by Anhalt et al. in 1990, who initially proposed a set of diagnostic criteria [2]. PNP-associated neoplasms have been frequently documented in a diverse range of hematological disorders, including Castleman disease, non-Hodgkin lymphoma, both malignant and benign thymomas, follicular dendritic cell sarcoma, macroglobulinemia, and chronic lymphocytic leukemia. Notably, these neoplasms have also been identified in various solid tumors, particularly in gastric, pulmonary, and colorectal carcinomas [3,4].

The diagnosis and management of PNP present challenges due to its highly variable clinical manifestation and frequent association with malignancies, compounded by the lack of standardized diagnostic criteria and established treatment protocols [1]. The diagnostic process is further complicated by the polymorphic nature of cutaneous presentations, necessitating a comprehensive approach that integrates detailed clinical evaluation with characteristic histopathological, immunopathological, and serological findings [5]. We herein report a rare case of PNP associated with pheochromocytoma.

## Case presentation

Patient information

**Figure 1:**
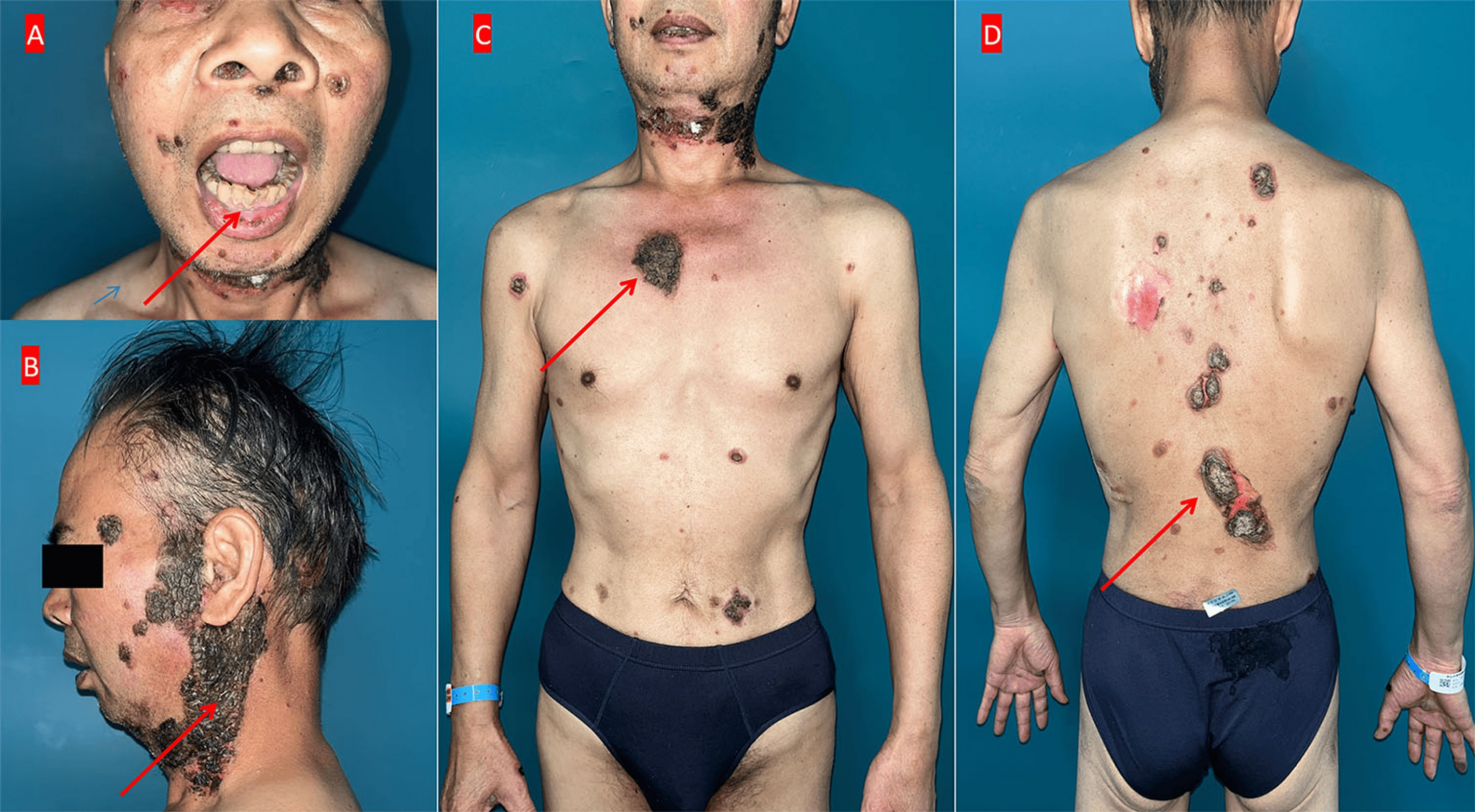
Physical examination revealing erosive blisters and scabs (arrows). A) face; B) neck; C) chest; D) back Written informed consent has been provided by the patient to have the case details and any accompanying images published.

A 62-year-old male presented with a progressive dermatological condition that initially manifested in July 2023. The patient first developed erythematous lesions accompanied by vesicles and bullae on the facial region. These cutaneous manifestations were characterized by fragile vesicles that readily ruptured, resulting in painful, pruritic erosions with associated exudation and crust formation. The patient initially neglected medical consultation until approximately one month post-onset, when he developed oral mucosal erosions. Despite seeking treatment at an external clinic, no clinical improvement was observed. The condition progressively worsened, with skin lesions extending to involve the trunk and extremities while maintaining facial involvement. Concurrently, the patient developed erosive lesions affecting multiple mucosal surfaces, including the conjunctiva, urethra, and nasal mucosa (Figure 1). Additionally, the patient had an abdominal mass for five years, which had remained untreated throughout this period. During the diagnostic workup one week prior to admission in March 2024, abdominal ultrasonography performed at an external outpatient facility revealed a mass localized to the pancreatic head and duodenum. Based on the clinical presentation and imaging findings, the patient was referred to our department for comprehensive evaluation and management. He was subsequently admitted on March 22, 2024, with a provisional diagnosis of PNP.

The patient had a 10-year history of diabetes mellitus, which had been managed with metformin at a dosage of 0.5 g daily, resulting in fasting blood glucose levels consistently maintained between 7 and 8 mmol/L. The abdominal mass caused no significant discomfort for five years. The patient had no family history of malignant tumors or pemphigus and no history of smoking.

Physical presentation, dermatological findings, and treatment

Upon admission, the patient's blood pressure fluctuated between 140 and 160/60-100 mmHg, heart rate fluctuated between 70 and 110 beats per minute, and other vital signs remained stable. The cardiopulmonary examination was unremarkable, and an abdominal mass could be palpated. Multiple erosions were exhibited on the right conjunctiva, nasal mucosa, oral cavity, and lip mucosa, accompanied by purulent discharge. Additionally, numerous erythematous lesions, erosions, vesicles, and bullae were observed on the scalp, face, neck, chest, abdomen, and back, accompanied by a positive Nikolsky sign. The bullae walls were loose and contained clear fluid. Most of the erosive surfaces displayed crusting that resembled oyster shells (Figure 1). Blood blisters were notably present on the palms and soles, particularly in the nail folds. A biopsy was performed on the skin lesion on the back, and plasma was extracted to detect antibodies for pemphigus. We initially used methylprednisolone (60 mg daily) and methotrexate (10 mg weekly) to treat pemphigus; however, the patient's erythema and vesicles regressed slowly, and new vesicles continued to appear, suggesting treatment resistance. Subsequently, we discontinued methotrexate and switched to rituximab injections (500 mg weekly for two doses, administered on April 4, 2024, and April 11, 2024), and the patient's skin lesions gradually became stable and dry.

**Figure 2:**
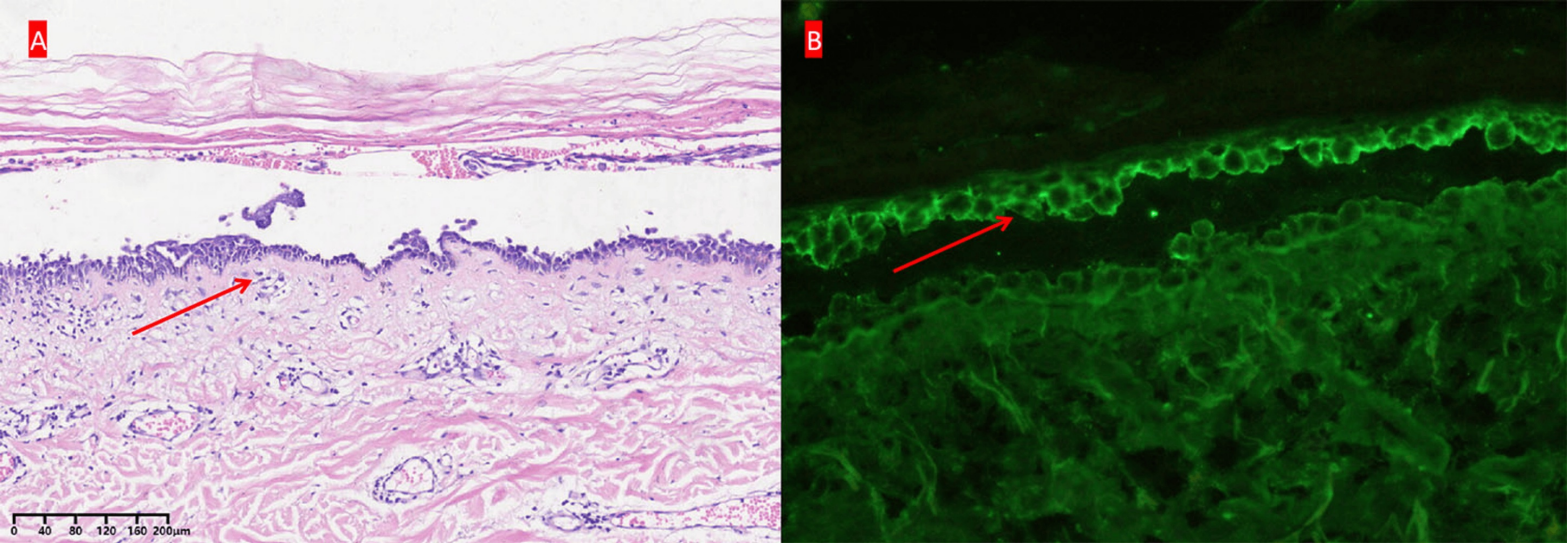
Histopathological examination of the abdominal skin biopsy (arrows). A) Histopathological examination, showing parakeratosis and crust formation, with scattered necrotic keratinocytes, liquefaction degeneration of basal cells, and infiltration of lymphocytes in the superficial dermis (H&E staining, x200); B) Direct immunofluorescence showing intercellular deposition with IgG positive reactivity.

**Figure 3:**
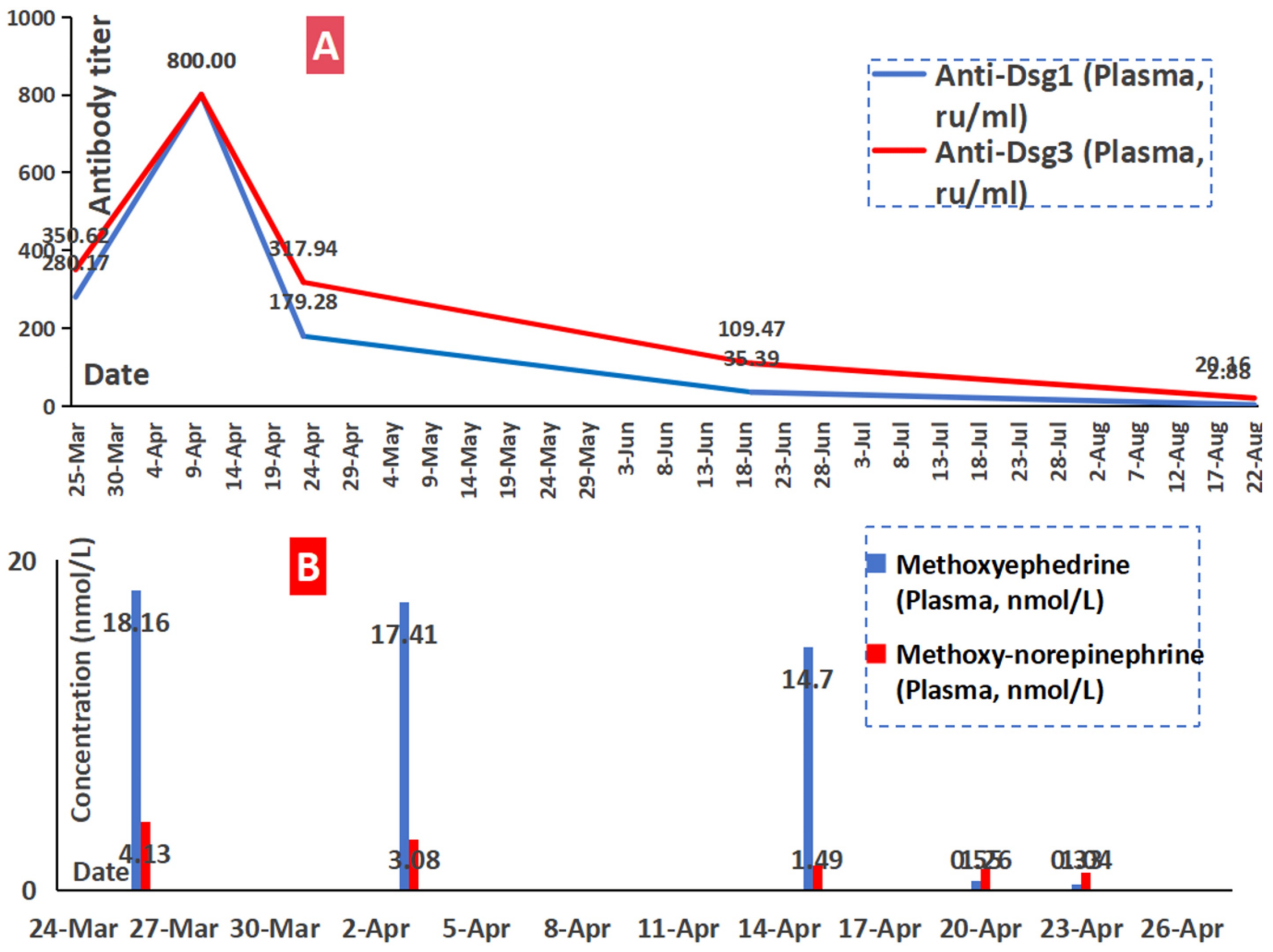
Clinical detection in plasma. A) Both titers of anti-Dsg1 and anti-Dsg3 in plasma showing a gradual decrease in both antibodies since the clinical management, including the laparoscopic resection on April 18, 2024. B) Both levels of methoxyephedrine and methoxy-norepinephrine in plasma revealing a decrease, especially in methoxyephedrine since the laparoscopic resection. Dsg: desmoglein

Histopathological examination of the back skin demonstrated characteristic features of parakeratosis and crust formation, accompanied by scattered necrotic keratinocytes and liquefactive degeneration of basal cells. The superficial dermis exhibited significant lymphocytic infiltration (Figure 2A). Immunohistochemical analysis using direct immunofluorescence (DIF) revealed positive staining for IgG and C3 along the dermoepidermal junction (Figure 2B), while IgM and IgA staining were negative. Based on clinical presentation, skin biopsy, and positive anti-desmoglein (Dsg) antibodies (Figure 3A), a diagnosis of pemphigus was confirmed.

Tumor imaging and surgical and perioperative management

**Figure 4:**
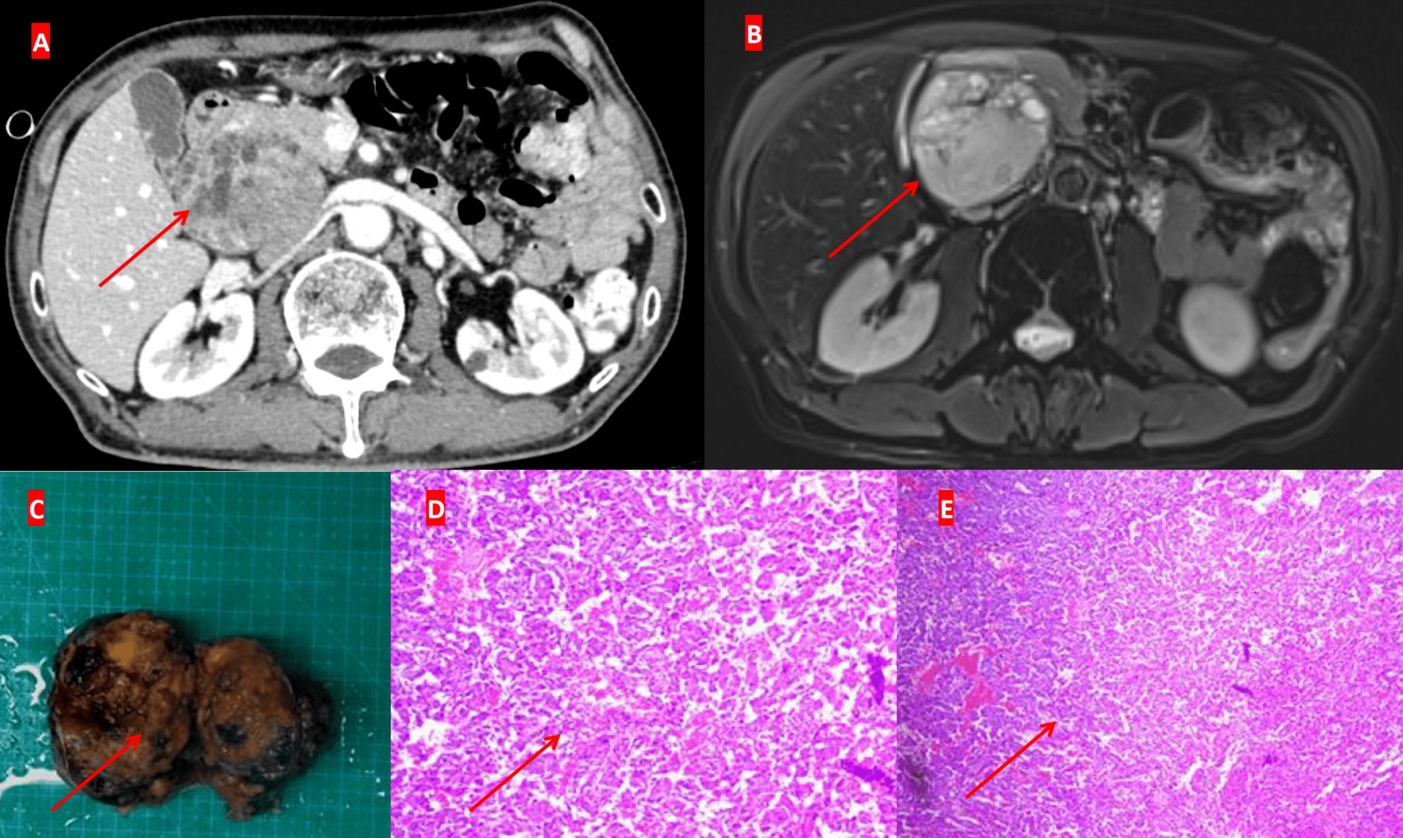
Retroperitoneal mass and examination (arrows). A) Computed tomography (CT), showing the mass; B) Magnetic resonance imaging (MRI), showing the solitary mass; C) Retroperitoneal mass, approximately 6 x 5 x 2.5 cm^3^; D) Histopathological examination (HE staining, x200), showing atypical cells with partial cellular atypia; E) Histopathological examination (HE staining, x40).

The abdominal mass was subjected to a comprehensive diagnostic workup to screen for possible tumors. Various tumor biomarkers, including carcinoembryonic antigen and alpha-fetoprotein, were essentially normal. After contrast-enhanced MRI and CT examinations, a well-defined solid mass in the duodenal groove area was confirmed, measuring approximately 65 mm × 52 mm × 48 mm, with consideration that originated from mesenchymal tissue (Figures 4A-4B). Following a consultation with the endocrine specialists, paraganglioma was considered a potential diagnosis, while plasma levels of methoxyephedrine, methoxy-norepinephrine, and urinary vanillylmandelic acid excretion were significantly elevated (Figure 3B).

Preoperative management included a two-week course of phenoxybenzamine therapy. Additionally, a three-day (April 16-18, 2024) perioperative glucocorticoid regimen (methylprednisolone daily, with a maximum dose of 60 mg) was implemented, consisting of intravenous administration of 20 mg of glucocorticoids daily during both the preoperative and intraoperative periods.

On April 18, 2024, the patient underwent a laparoscopic resection of a left retroperitoneal mass under general anesthesia, revealing a mass measuring 6 × 5 × 2.5 cm³ (Figure 4C), for which a pathological examination was subsequently performed.

Postoperatively, he was transferred to the ICU for monitoring and received supportive care with antibiotics. On April 21, 2024, he continued to receive 20 mg of methylprednisolone, while pathological examination revealed a diagnosis of pheochromocytoma, presenting partial cellular atypia in atypical cells (Figures 4D-4E).

On April 20 and 23, 2024, follow-up laboratory tests demonstrated a significant reduction in both blood and urine levels of metanephrines, including methoxyphenamine and methoxy-norepinephrine. Concurrently, antibody titers against desmoglein-1 (Dsg1) and desmoglein-3 (Dsg3) also showed a decreasing trend (Figure 3B). Since then, the patient has demonstrated significant clinical improvement, characterized by the absence of new bullae formation and progressive healing of both mucosal and cutaneous erosions (Table 1), leading to his discharge on the morning of April 30, 2024.

**Table 1:** Clinical events related to the examination, treatment, and presentation improvement. Dsg: desmoglein

Time/event	Laboratory finding	Treatment	Clinical finding
March 22, 2024, admission	Anti-Dsg1 and anti-Dsg3 (+), MRI and CT showing a solid mass	1. Methylprednisolone (60 mg qd); 2. Methotrexate (10 mg qw)	Multiple erosions on body and mucosa
April 4-11, 2024	-	1. Methotrexate discontinued; 2. Rituximab injections (500 mg, 2 doses/week)	Skin lesions gradually improving after rituximab
Preoperative	Methoxyephedrine and methoxy-norepinephrine in plasma significantly elevated and vanillylmandelic acid in urine significantly elevated	1. Phenoxybenzamine for two weeks (20 mg bid); 2. Immunoglobulin (20 g qd) for three days (April 16-18, 2024)	The possibility of a paraganglioma in the duodenal groove area was considered
April 18, 2024	The mass was 6 x 5 x 2.5 cm³; pathologically confirmed as a pheochromocytoma	Under general anesthesia; 20 mg of methylprednisolone and antibiotics daily	Laparoscopic resection of a retroperitoneal mass
April 30, 2024, discharge	Methoxyephedrine and methoxy-norepinephrine in plasma significantly reduced	Medications after discharge: 1. Methylprednisolone (20 mg qd); 2. Methotrexate (10 mg qw)	No new bullae formation; mucosal and cutaneous erosions healed
June 19, 2024	Anti-Dsg1 and anti-Dsg3 significantly reduced	1. Methylprednisolone (20 mg qd); 2. Methotrexate (10 mg qw)	No observable erosion
August 22, 2024	Anti-Dsg antibodies decreased to near-normal levels	1. Methylprednisolone (12 mg qd); 2. Methotrexate (10 mg qw)	-
October 14, 2024	-	1. Methylprednisolone (8 mg qd); 2. Methotrexate (5 mg qw)	Skin lesions mostly healed

Out-patient follow-up

Follow-up observations revealed the following clinical course: On May 8, 2024, the cutaneous manifestations remained stable, with residual erosions sporadically visible on the jaw and back, while the scalp showed complete scab formation. By June 19, 2024, the rash had stabilized further, with no observable erosions on the body, though two pustules were noted on the left temporal scalp. Subsequent evaluations on August 22, 2024, and October 13, 2024, showed scattered papulopustules in the mandibular region without any evidence of cutaneous erosions. Throughout this period, oral low-dose methylprednisolone therapy was maintained as part of the ongoing management. A decrease in pemphigus-associated antibody titers was observed during outpatient follow-up (Figure 3A).

## Discussion

PNP is a form of pemphigus that develops in association with tumors, representing approximately 5% of all pemphigus cases, while PNP is more commonly linked with lymphoma, especially non-Hodgkin lymphomas, and is rarely reported in association with pheochromocytoma. This condition predominantly manifests in adults between 45 and 70 years of age, with relatively equal sex distribution (13 cases in males vs. 14 cases in females) [6-8]. This report presents the case of a 62-year-old male patient diagnosed with PNP, which manifests as a rare dermatological disorder. The patient has no family history of the disease and no relevant exposure history (such as special medications, infections, stress, diet, and so on).

Currently, there is a lack of universally accepted standardized diagnostic criteria for PNP [4]. However, the previous study proposed diagnostic criteria comprising three major and two minor components [1]. The major components include (a) the presence of mucous membrane lesions, with or without cutaneous involvement; (b) the coexistence of an internal neoplasm; and (c) serological evidence of anti-plakin antibodies. The minor components comprise (a) histopathological findings of acantholysis and/or lichenoid interface dermatitis and (b) DIF staining revealing intercellular and/or basement membrane staining. A definitive diagnosis can be made when either all three major criteria or two major criteria plus both minor criteria are met. As to this case, it fulfilled the diagnostic components, as evidenced by the presence of mucocutaneous lesions, internal neoplasm, and histopathological and immunohistochemical findings. Certainly, pheochromocytoma represents a distinct clinical entity that warrants comprehensive diagnostic evaluation [5].

The characteristics of this case include multiple skin lesions on the face, neck, abdomen, and back, along with mild lesions on the oral mucosa, while the skin biopsy showed suprabasal epithelial detachment with a lymphocytic and neutrophilic infiltrate, and DIF showed positive fluorescence in the intercellular cement substance (ICS) of IgG and complement C3, with negative IgA and IgM. Compared with these, the previous cases of PNP with prostate cancer showed oral erosive mucositis, lips, and endonasal crusted erosions with positive IgG of DIF, or plus positive enzyme-linked immunosorbent assay (ELISA) of autoantibody [9,10]. This patient's clinical condition showed gradual improvement after surgical treatment and medication, demonstrating the efficacy of therapeutic management. The case will be followed up in the future.

Histopathological examination of this pheochromocytoma revealed the presence of atypical cells with partial cellular atypia, suggesting a transitional state toward malignant transformation. Following treatment with rituximab, methotrexate, and methylprednisolone, the patient's symptoms gradually improved. Concurrently, both anti-Dsg1 and anti-Dsg3 antibody levels, as well as blood methoxyephedrine, decreased, suggesting that rituximab, in combination with other agents, was an effective therapeutic option for treating pemphigus [11].

Pheochromocytomas originate from chromaffin cells located in the adrenal medulla, developing from the extra-adrenal sympathetic and parasympathetic ganglia, which account for 80% to 85% of paragangliomas (PPGLs) cases [12]. PPGLs represent a rare category of neuroendocrine tumors, characterized by their distinct origins: 80-85% arise from the adrenal medulla, while the remaining 15-20% develop from extra-adrenal chromaffin tissues. Notably, approximately 30-40% of PPGL cases exhibit a hereditary predisposition, positioning them among the tumor types with the highest genetic susceptibility [12].

Genetic research has classified PPGLs into three distinct molecular clusters based on their underlying pathogenic mechanisms: pseudohypoxia-related, kinase-signaling, and WNT-signaling pathway variants [13]. Like studying infectious diseases, genetic research might be an important approach to exploring the autoimmune etiology and prevention of pemphigus and PNP [14]. The PNP in this case might represent a distinctive clinical feature among pheochromocytomas, suggesting a potential novel subtype that warrants further investigation through comprehensive genetic analysis and classification.

## Conclusions

PNP with pheochromocytoma is a rare yet significant condition that may present with complex symptoms. Early identification and treatment of the underlying malignancy are crucial for management and improving patient prognosis, warranting further research from both autoimmune and biogenetic perspectives.
